# Versican Proteolysis by ADAMTS: Understanding Versikine Expression in Canine Spontaneous Mammary Carcinomas

**DOI:** 10.3390/cancers16234057

**Published:** 2024-12-04

**Authors:** Maria Carolina Souza, Simone Nunes, Samantha Hellen Santos Figuerêdo, Bruno Sousa de Almeida, Isac Patrick Conceição Santos, Geovanni Dantas Cassali, Sérgio Marcos Arruda, Thiago Marconi de Souza Cardoso, Alessandra Estrela-Lima, Karine Araújo Damasceno

**Affiliations:** 1 Laboratory of Experimental Pathology, Gonçalo Moniz Institute, Oswaldo Cruz Foundation, 121 Rua Waldemar Falcão, Salvador 40296-710, BA, Brazil; mariacarolina.medvet@gmail.com (M.C.S.); simonenunes.vet@gmail.com (S.N.); samanthahellen_95@hotmail.com (S.H.S.F.); almeidabrsousa@gmail.com (B.S.d.A.); citologistaisac.p@outlook.com (I.P.C.S.); 2 Comparative Pathology Laboratory, Department of Pathology, Institute of Biological Sciences, Federal University of Minas Gerais, 6627 Av. Pres. Antônio Carlos, Belo Horizonte 31270-901, MG, Brazil; geovanni.cassali@gmail.com; 3 Advanced Public Health Laboratory, Gonçalo Moniz Institute, Oswaldo Cruz Foundation, 121 Rua Waldemar Falcão, Salvador 40296-710, BA, Brazil; sergio.arruda@fiocruz.br; 4 Clinical Research Laboratory, Gonçalo Moniz Institute, Oswaldo Cruz Foundation, 121 Rua Waldemar Falcão, Salvador 40296-710, BA, Brazil; thiago.cardoso@fiocruz.br; 5 Research Center on Mammary Oncology NPqOM/HOSPMEV, Federal University of Bahia, Salvador 40170-110, BA, Brazil; aestrela@ufba.br

**Keywords:** mammary cancer, dogs, extracellular matrix, metalloproteinase, proteoglycan

## Abstract

**Background:** The present study investigates VKINE, a bioactive proteolytic fragment of the proteoglycan VCAN, as a novel and significant element in the tumor extracellular matrix (ECM). Although VKINE has been recognized for its immunomodulatory potential in certain tumor types, its impact on ECM degradation and prognostic implications remains poorly understood. **Objectives**: This study aimed to evaluate VCAN proteolysis and its association with ADAMTS enzymes involved in extracellular matrix remodeling in spontaneous canine mammary gland cancer. **Methods**: The expression levels of VKINE, ADAMTS enzymes, and collagen fibers were comparatively analyzed in situ and in invasive areas of carcinoma in mixed tumor (CMT) and carcinosarcoma (CSS) with different prognoses. **Results**: VKINE was notably expressed in the stroma adjacent to the invasion areas in CMT, whereas ADAMTS-15 was identified as the enzyme associated with VCAN proteolysis. Inverse correlations were observed between type III collagen and VCAN expression in in situ areas. **Conclusions**: Our findings suggest that VKINE and ADAMTS-15 play crucial roles in the tumor microenvironment, influencing invasiveness and type III collagen deposition. This study contributes to a better understanding of the dynamics within the ECM, paving the way for potential new tools in diagnosing and treating human and canine mammary tumors.

## Simple Summary

A tumor’s extracellular matrix (ECM) serves as a vital connection and support network, orchestrating events that regulate tumor activity. Versican (VCAN), a proteoglycan found within the ECM, plays a crucial role in invasion and metastasis. Like the ECM, VCAN can undergo cleavage by metalloproteinases, such as a disintegrin and metalloproteinase with thrombospondin motifs (ADAMTS). This proteolytic process produces a bioactive fragment known as versikine (VKINE). Remarkably, this fragment exhibits immunomodulatory activity in certain cancer types. Consequently, the proteolysis of VCAN and associated factors may be valuable indicators of tumor progression.

## 1. Introduction

Among the various lines of cancer research, studies focusing on the tumor microenvironment, particularly the dynamics of the extracellular matrix (ECM) and its components, have yielded promising data. These findings are crucial for identifying new biomarkers for early diagnosis, prognosis, and therapeutic targets. They also enhance our understanding of the mechanisms underlying tumor invasion and metastasis in both female dogs and women [1,2,3,4,5,6].

In this regard, the proteoglycan (PG) VCAN, a component of the ECM, has garnered significant interest among researchers over the past few decades. Many studies have shown that VCAN has anti-adhesive properties, the ability to modulate proliferation, cell migration, and angiogenesis, and is associated with the invasiveness potential of malignant tumors, thereby contributing to tumor progression [7,8,9,10].

VCAN and other hyalectan proteoglycans, fundamental components of the extracellular matrix, are directly regulated by ADAMTS (a disintegrin and metalloproteinase with thrombospondin motifs). Under physiological conditions, there is a balance between ADAMTS enzymes and proteoglycans; however, studies provide strong evidence that, in the tumor microenvironment, an imbalance between them may either promote tumor progression or stimulate antitumor mechanisms in a context-dependent manner [11,12,13].

ADAMTS enzymes belong to the metzincin superfamily, which represents a large group of zinc-dependent metalloproteinases. The ADAMTS family consists of 19 members, all of which are characterized by an N-terminal protease region containing a pro-domain, a metalloproteinase domain, and a disintegrin-like domain [14,15,16]. These enzymes can perform different activities. For example, ADAMTS-1, -4, -5, -8, -9, -15, and -20 predominantly exhibit proteoglycanase activity, with VCAN and ACAN as their main substrates in various tumor types (pancreatic, hepatocellular, brain, melanoma, prostate, and breast), as well as in extracellular matrix remodeling, proliferation, and morphogenesis in embryonic tissue [11,17].

VCAN, when cleaved by ADAMTS enzymes, results in the formation of VKINE, a bioactive proteolytic fragment. VKINE was recently identified as an important matrikine with immunomodulatory potential in some tumor types, including multiple myeloma and human colorectal cancer [18,19,20,21,22]. However, the role of VCAN proteolysis in the modulation of inflammatory cells within the tumor microenvironment, as well as its influence on ECM degradation and potential prognostic significance, remains poorly investigated [19].

In the context of women’s breast cancer, VKINE still has not been thoroughly investigated. Breast cancer is currently the most frequent neoplasm worldwide, with an estimated 73,610 new cases per year in Brazil during the 2023–2025 triennium [23]. Dogs are the only species with a comparable prevalence of mammary tumors, presenting a significant challenge in veterinary practice and leading to the death of many companion animals, which are considered important members of multispecies families in modern times. VKINE has not been studied in canine mammary cancer. Due to the similarities between humans and canines, canine mammary tumors are a valuable model for studying VCAN proteolysis in tumor microenvironments. Likewise, canine mammary cancer shares several aspects with human cancer [24,25,26], making it an interesting model for breast cancer research. The rapid progression of the disease in canines provides an advantage for obtaining results applicable to both species [27,28,29].

Our group previously described VCAN overexpression in the peritumoral stroma adjacent to invasive areas of benign mixed tumors, CMT, and CSS [30]. Based on these previous results, we conducted the first investigation into VCAN proteolysis and its association with ADAMTS enzymes involved in extracellular matrix remodeling in spontaneous canine mammary gland cancer. The purpose of this work is to contribute to a better understanding of extracellular matrix remodeling during cancer progression and, in the future, to aid in identifying new therapeutic targets for both human and canine mammary tumors.

## 2. Materials and Methods

### 2.1. Case Selection

A total of 30 dogs with a histopathological diagnosis of CMT and 17 with CSS were selected. The patients were female dogs of any breed or age, either intact or sterilized, diagnosed with a mammary tumor, which had undergone a unilateral radical mastectomy between 2018 and 2019 by the mammary cancer oncology research group at the Hospital of Veterinary Medicine Professor Renato Medeiros Neto (HOSPMEV/UFBA), Salvador, Bahia, Brazil. Primary mammary tumors were collected from these patients.

### 2.2. Anatomopathological Study

The anatomopathological study was conducted at the Experimental Pathology Laboratory of the Gonçalo Moniz Institute (*Instituto Gonçalo Moniz*), Salvador, Brazil. For histopathological analysis, 4 µm histological sections were obtained from the mammary gland fragments processed using the routine paraffin embedding technique and stained with hematoxylin–eosin. The identification of the histological type followed the Consensus for the Diagnosis, Prognosis, and Treatment of Canine Mammary Tumors [31] performed by a veterinary pathologist specializing in mammary oncology. The *in situ* areas were defined by observing epithelial cells in a tubular arrangement with intact myoepithelial cell layers and basal membrane, as shown by hematoxylin–eosin staining [31].

### 2.3. Immunohistochemistry

The primary antibodies used in the immunohistochemical analysis were VCAN (1:50, clone 12C5, DSHB, Iowa City, IA, USA), VKINE (neo-epitope DPEAAE, 1:500, polyclonal, ThermoFisher Scientific, Waltham, MA, USA), ADAMTS1 (1:80 clone 3C8F4, Santa Cruz, Dallas, TX, USA), ADAMTS-5 (1:100, clone Ab41037, Abcam, Cambridge, UK), ADAMTS8 (1:100, clone 31G7, Invitrogen, Vacaville, CA, USA), ADAMTS-9 (1:1000, polyclonal, Invitrogen, Vacaville, CA, USA), and ADAMTS-15 (1:500, clone 561819, Invitrogen). To validate the use of anti-VKINE and ADAMTS antibodies, the sequence homology of the target human and canine antigens was compared using the Basic Local Alignment Search Tool (BLAST). The homology of all markers exceeded 80%, and the antibodies were, therefore, considered suitable for use.

For the immunohistochemistry technique, 4 μm sections were cut from one representative block of each case and collected on gelatinized slides. Tissue sections were deparaffinized, rehydrated in a graded ethanol series, and subjected to heat-induced antigen retrieval. A pressure cooker at 114 °C (70 kPa) for 20 min and 10 mM citrate buffer (pH 6.0) was used for antigen retrieval of all antibodies except VKINE and VCAN. For VKINE antigen retrieval, citrate-EDTA antigen retrieval buffer (10 mM citric acid, 2 mM EDTA, 0.05%, pH 6.2) was used. The sections were placed in a microwave, heated at 500 W for 90 s, and left to stand for 30 s, and the heating step was repeated [32]. For VCAN, enzymatic retrieval was performed using 0.5 U/mL chondroitinase ABC (*Proteus vulgaris*; Sigma Chemicals, St. Louis, MO, USA) in 0.25 M Tris buffer (pH 8.0) with 0.18 M sodium chloride and 0.05% bovine serum albumin (BSA) at 37 °C for 1 h and 30 min. A 0.25 M Tris buffer solution (pH 8.0) with 0.1 M 6-amino-n-caproic acid and 5 mM benzamidine hydrochloride was used for 30 min to inhibit protease activity ([33] adapted).

Next, polymerization was performed, and antigens were identified using secondary antibodies (ADVANCE HRP—ready to use—DakoCytomation, Glostrup, Denmark). Diaminobenzidine was used as the chromogen, and the sections were counterstained with Mayer’s hematoxylin, hydrated, and mounted in a synthetic medium. Negative controls were prepared by replacing the primary antibody with normal serum. Canine mammary tumors previously known to express VCAN and tissue with abundant myxoid matrix expressing VCAN were used as positive controls.

### 2.4. Immunohistochemical Evaluation

The VCAN and the VKINE expression were evaluated in the cytoplasm of epithelial and myoepithelial cells, as well as in the stroma of areas adjacent to malignant in situ or invasive epithelial proliferation. This evaluation was conducted using the semiquantitative scoring system proposed by SKANDALIS and collaborators [34].

The analysis of the markers was performed at 20× magnification and confirmed at 40× magnification by two pathologists in a double-blind format, and the staining intensity was evaluated on a scale from 0 to 4. According to the scale, (0) is considered negative, (1) weak positive, (2) moderate positive, and (3) strong positive. In the stroma, the degree of VCAN and VKINE expression was determined by multiplying the percentage (0–100%) by the intensity (0–3) [8]. For VCAN and VKINE, cells showing positive marking for ADAMTS enzymes were classified according to the area of analysis (in situ or invasive, stromal or epithelial) and according to the expression score obtained from multiplying the intensity (0–3) by the extent of the area marked in percentage (0–100%) ([35] adapted). Slides that demonstrated any technical artifact that could interfere with the analysis were excluded from this study.

### 2.5. Special Stains and Histomorphometry

The sections submitted to immunohistochemistry were also subjected to special stains to analyze and characterize the degradation of the ECM in the studied samples. Periodic acid–Schiff (PAS) was used to assess the integrity of the basement membranes [36], thus helping to identify areas in situ.

Masson’s Trichrome was used to measure the collagen present in the invasion areas, in situ injury, and total collagen in the histological section [37]. Picrosirius Red staining was used to characterize collagen as type I or III under polarized light [38,39,40]. In these stains, the blue pixels (Masson’s Trichrome) and green or red pixels (Picrosirius Red) were quantified using a standardized semi-automatic method developed in our laboratory using the ImageJ software, Fiji version 2.9.0 (Versatile tool).

### 2.6. Statistical Analysis

The analyses were performed using the statistical software GraphPad Instat v. 8.0.1 (GraphPad, San Diego, CA, USA). The normality of the data was assessed using the Shapiro–Wilk test. The quantitative results that showed a normal distribution were subjected to analysis of variance (ANOVA) at 5% probability, followed by the means test. Differences between CMT and CSS were analyzed using the Mann–Whitney test or the *t*-test. Differences between in situ and invasive areas were analyzed using the Wilcoxon Test used for non-parametric data. Correlations were assessed using Spearman’s test for non-parametric data. Values were considered statistically significant when *p* < 0.005.

### 2.7. Ethic Aspects

This study was approved by the Animal Use Ethics Committee of the Gonçalo Moniz Institute, protocol number 07/2018.

## 3. Results

### 3.1. Evaluation of the Expression Profile of VCAN and VKINE in CMT and CSS

**Figure 1:**
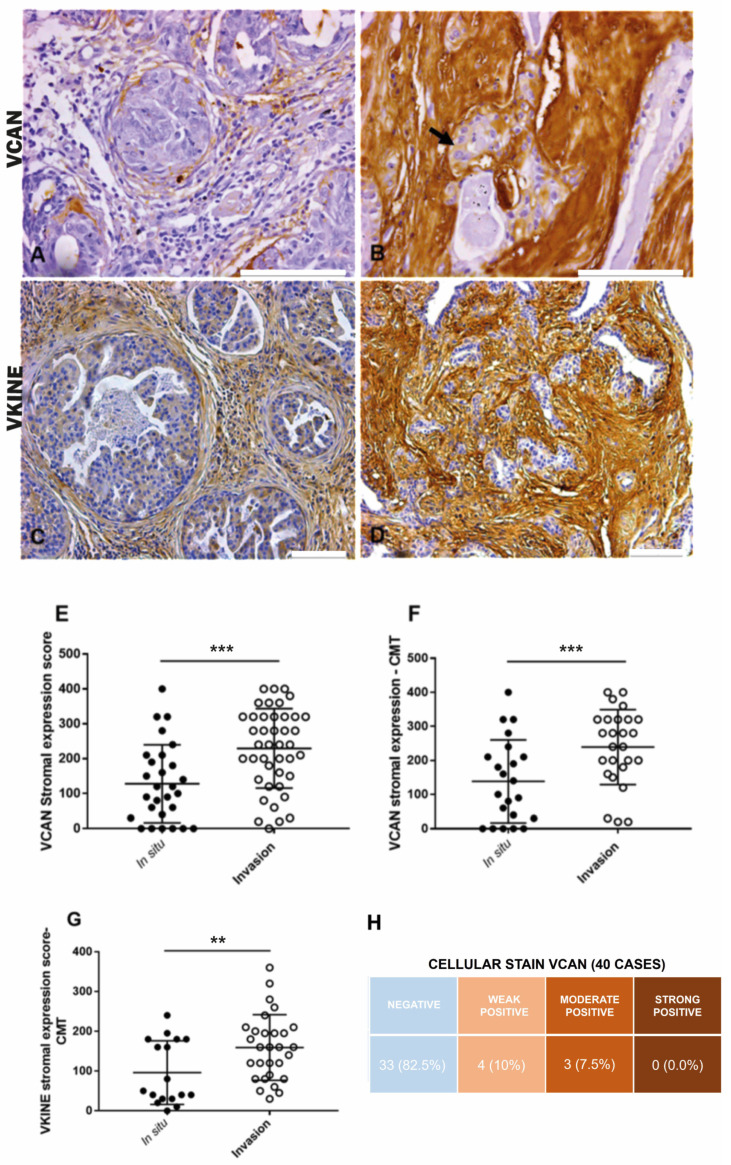
Immunohistochemical VCAN and VKINE expressions. (**A**) Moderate positive expression of VCAN in the stroma adjacent to carcinomatous areas in situ in CMT, 400×, bar 100 µm. (**B**) Moderate positive cytoplasmic expression of VCAN in carcinoma cells (black arrow) and strong expression in the stroma adjacent to areas of invasion in CMT, 400×, bar 100 µm. (**C**) Moderate positive expression of VKINE in the in situ area of CMT, 200×, bar 100 µm. (**D**) Strong positive expression of VKINE in the area of invasion, 200×, bar 100 µm. (**A**–**D**) Counterstaining with Mayer’s hematoxylin. (**E**) Difference in VCAN expression in stroma between in situ and invasive carcinomatous in CTM types (*** *p* ≤ 0.0001, Wilcoxon test). (**F**) Difference in VCAN expression in the stroma between in situ and invasion areas in CMT (*** *p* ≤ 0.0001, Wilcoxon test). (**G**) Difference in VKINE expression in the stroma between in situ and invasive carcinomatous areas in CMT (** *p* ≤ 0.0048, Wilcoxon test). (**H**) Intensity of VCAN staining in carcinoma cells in the analyzed cases. Primary antibody used: VCAN (1:50, clone 12C5, DSHB, Iowa City, IA, USA). *p*-values ≤ 0.05 were considered significant.

In CMT, VCAN expression was higher in the stroma adjacent to invasion areas (median: 100.0) compared to in situ areas (median: 225.0) (*p* ≤ 0.0001) (Figure 1A,B,E). Overall, in both CMT (*p* ≤ 0.0001) and CSS (*p* = 0.03), VCAN was more prevalent in the stroma of carcinomatous invasion areas, which exhibited larger areas of strong VCAN expression (Figure 1F). There was no significant difference in VCAN expression between in situ and invasion areas in the CSS samples. Cytoplasmic staining in carcinomatous cells in both CMT and CSS (Figure 1B) was observed in seven cases, with three cases showing moderate expression and four cases showing weak expression. The data are presented in Figure 1H.

**Figure 2:**
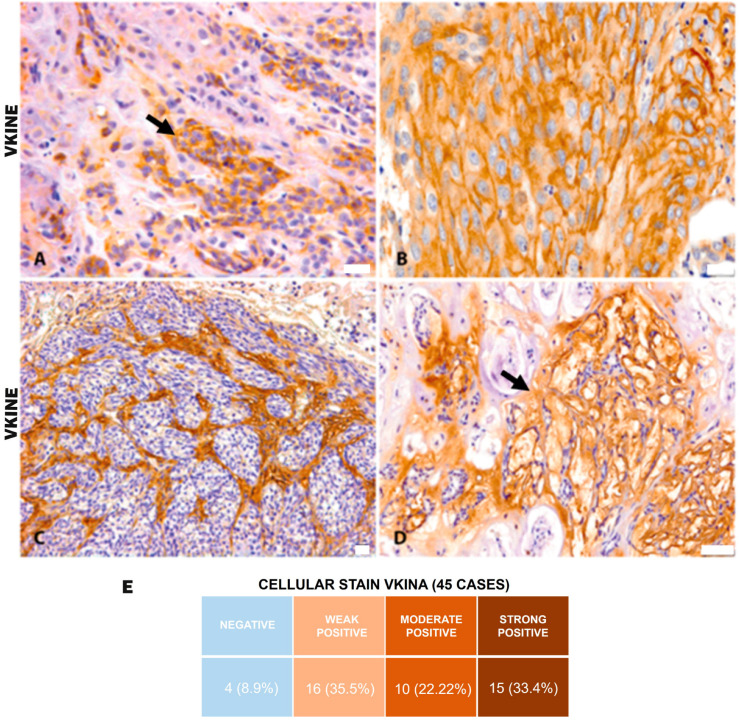
VKINE immunostaining in canine mammary tumors. (**A**) VKINE expression in the cytoplasm in carcinosarcoma (black arrow), 400×, bar 20 µm. (**B**) VKINE labeling on epithelial cell membrane with squamous differentiation into mixed tumor carcinoma, 400×, bar 20 µm. (**C**) VKINE expression in stroma adjacent to carcinomatous invasion areas in carcinosarcoma, 200×, bar 20 µm. (**D**) VKINE expression in a myxoid matrix in CMT (black arrow), 200×, bar 50 µm. (**A**–**D**) Counterstaining with Mayer’s hematoxylin. (**E**) Table illustrating the number of cases with cellular stain according to intensity. Primary antibody used: VKINE (neo-epitope DPEAAE, polyclonal, ThermoFisher Scientific, Waltham, MA, USA).

All analyzed cases were positive for VKINE expression, indicating that all samples showed VCAN proteolysis. Three types of staining were observed in the studied samples: cytoplasmic, membrane, and stromal (Figure 2A–C). Strong VKINE immunoreactivity was also frequently found in the stroma of areas with myxoid matrix deposition (Figure 2D). Cytoplasmic expression in carcinomatous cells was present in most cases, varying from weak (16/45; 35.6%) to intense (15/45; 33.3%) (Figure 2E).

In CTM, VKINE expression was higher in the stroma adjacent to the invasion areas than in situ areas (*p* = 0.0048) (Figure 1C,D,G). Regarding CSS, no difference in VKINE expression was observed between in situ and invasion areas.

### 3.2. ADAMTS Enzymes and VKINE Production

**Figure 3:**
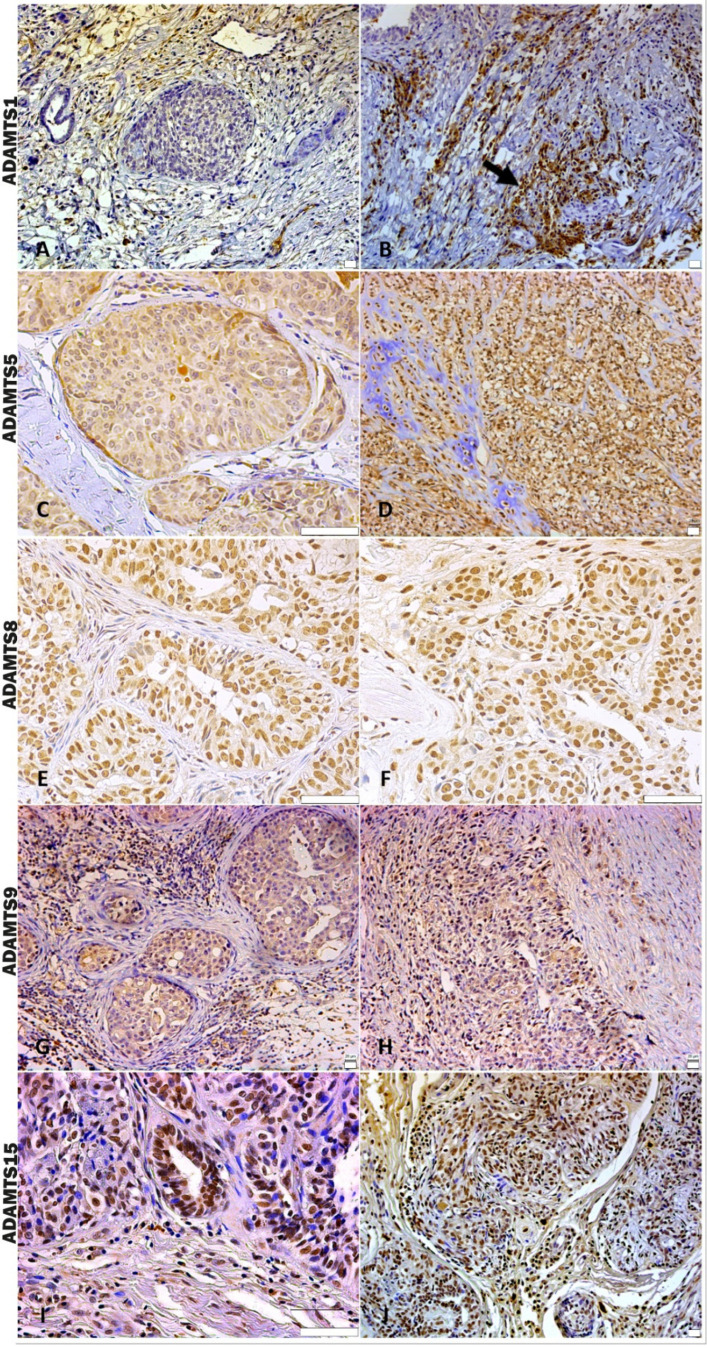
Immunostaining pattern of ADAMTS 1, 5, 8, 9, and 15. (**A**) Moderate positive ADAMTS-1 staining of stromal and inflammatory cells near the in situ area in CMT, 200×, bar 20 µm. (**B**) Strong positive cytoplasmic ADAMTS-1 staining of macrophages around invasive carcinoma cells (black arrow), 200×, bar 20 µm. (**C**) In situ area in CMT revealing predominant moderate positive cytoplasmic staining and absent or weak stromal ADAMTS-5 staining, 400×, bar 50 µm. (**D**) ADAMTS-5 in an area with chondromyxoid matrix in the CSS, showing moderate positive stromal and cellular staining, 200×, bar 20 µm. (**E**) In situ area of CMT showing expressive nuclear and weak cytoplasmatic ADAMTS-8 staining, 400×, bar 50 µm. (**F**) Invasive carcinomatous areas in CSS, nuclear staining pattern in ADAMTS-8 staining 400×, bar 50 µm. (**G**) In situ areas showing strong positive cytoplasmic ADAMTS-9 staining in carcinomatous and immune cells in CMT, 200×, bar 20 µm. (**H**) Area of invasion with strong positive cytoplasmic and stromal ADAMTS-9 staining in CMT, 200×, bar 20 µm. (**I**) Strong positive nuclear staining in carcinoma cells and moderate cytoplasmic immunolabeling in immune cells of ADAMTS-15 in CMT, 400×, bar 50 µm. (**J**) Strong positive nuclear and cytoplasmic staining in carcinoma cells and stromal staining of ADAMTS-15 in CMT, 200×, bar 20 µm. (**A**–**J**) Counterstaining with Mayer’s hematoxylin. Antibodies used: ADAMTS1 (clone 3C8F4, Santa Cruz, Dallas, TX, USA), ADAMTS-5 (clone Ab41037, Abcam, Cambridge, UK), ADAMTS8 (clone 31G7, Invitrogen, Vacaville, CA, USA), ADAMTS-9 (polyclonal, Invitrogen, Vacaville, CA, USA), and ADAMTS-15 (clone 561819, Invitrogen, Vacaville, CA, USA).

**Figure 4:**
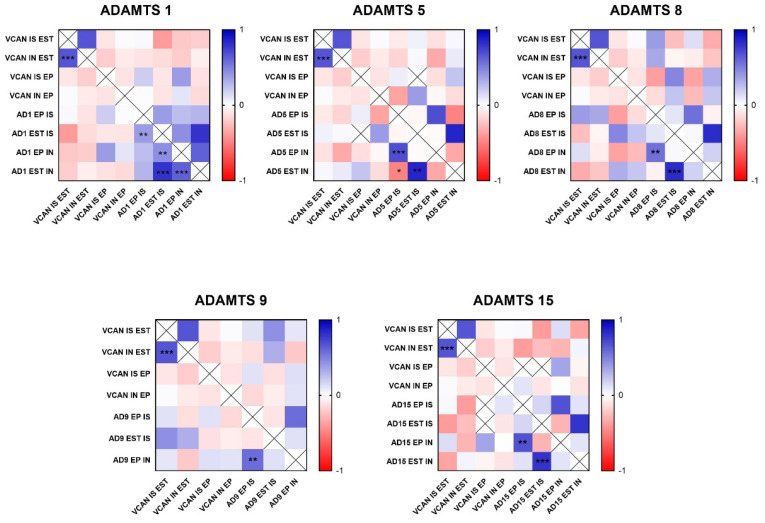
Heat map matrix of Spearman correlation analysis showing the correlation coefficients among ADAMTS enzymes and Versican expression in different areas in canine mammary tumors (in situ, invasion, epithelial, and stromal areas). Legend: AD = ADAMTS; VCAN = versican; IS = in situ; IN = invasion; EP = epithelial; EST = stromal; *p*-value: * = <0.05; ** = <0.01; *** = <0.001.

**Figure 5:**
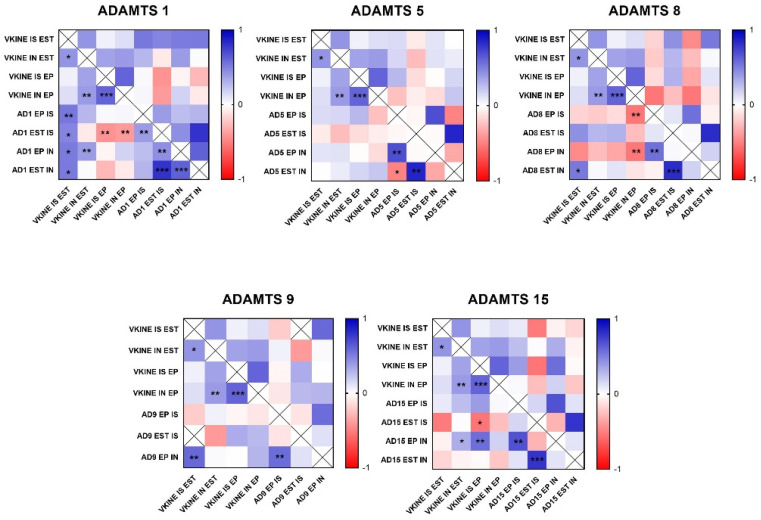
Heat map matrix of Spearman correlation analysis showing the correlation coefficients among ADAMTS enzymes and versikine expression in different areas in canine mammary tumors (in situ, invasion, epithelial, and stromal areas). Legend: AD = ADAMTS; VKINE = versikine; IS = in situ; IN = invasion; EP = epithelial; EST = stromal; *p*-value: * = <0.05; ** = <0.01; *** = <0.001.

In the analysis of ADAMTS-1 expression, some cases did not show significant reactivity (Table 1). In the positive samples, weak staining was observed in the cytoplasm of carcinomatous cells and moderate staining in macrophages in intratumoral stroma (Figure 3A,B). ADAMTS-1 did not show a significant correlation with VCAN expression (Figure 4); however, it did show a positive correlation in in situ areas (r = 0.4; *p* = 0.03) and invasive areas (r = 0.3; *p* = 0.01) with VKINE expression. A negative correlation was also observed between ADAMTS-1 expression in in situ stroma and VKINE expression in situ (r = −0.4; *p* = 0.03) and invasive carcinoma cells (r = −0.3; *p* = 0.01) (Figure 5).

The analysis of ADAMTS-5 revealed that the staining pattern in both histological types was predominantly cytoplasmic in both in situ (Figure 3C) and invasive areas (Figure 3D). However, compared to CSS, CMT revealed higher expression in invasive malignant epithelium (*p* = 0.040) and stroma (*p* = 0.099). In the in situ epithelium, CMT also showed a higher expression of ADAMTS-5 than CSS (*p* = 0.0592). This enzyme had no significant correlation with the VCAN expression and VKINE (Figure 4 and Figure 5).

Although ADAMTS-8 revealed a predominantly cytoplasmic expression profile in carcinoma cells, stromal expression adjacent to the evaluated *in situ* and invasion areas was also found (Figure 3E,F). CMT exhibited more ADAMTS-8 expressions than CSS in the in situ epithelium (*p* = 0.055). This enzyme showed a moderate negative correlation with the VKINE expression of carcinomatous cell invasion areas (r= −0.5; *p* = 0.002) (Figure 5) and had no significant correlation with VCAN.

ADAMTS-9 expression, in both in situ and invasive carcinomatous areas, showed predominant staining in epithelial cells, with only one sample displaying stromal staining (Figure 3G,H). Although with no statistical difference (*p* = 0.3690), ADAMTS-9 was more abundant in the invading epithelium of CMT (median = 60) than CSS (median= 30—Table S1). ADAMTS-9 did not show a significant correlation with VCAN; however, in invasion, it showed a positive correlation with VKINE expression in the stroma in situ (r = 0.5; *p* = 0.01).

The phenotype of cells expressing ADAMTS-15 predominantly showed nuclear staining; however, cytoplasmic staining in epithelial cells, as well as in stroma and inflammatory cells, such as lymphocytes and plasma cells, were also observed (Figure 3I,J). Among the enzymes with versicanase potential evaluated in this study, only ADAMTS-15 was differentially expressed between the high- and low-VKINE expression groups (*p* = 0.0355). In the Spearman correlation analysis, ADAMTS-15 expression in invasive areas was positively correlated with VKINE expression in invasive areas and in situ. In in situ areas, the expression of the enzyme in the stroma showed a negative correlation with the expression of VKINE in carcinoma cells (r = 0.5; *p* = 0.05) (Figure 5).

The median expression of ADAMTS-15 in stroma adjacent to in situ carcinomatous area was 120 in CSS and CMT, while in invasive areas, a median of 20 was obtained in this tumor type, despite there being no statistical difference (*p* = 0.7500) (Tables S1 and S2). The number of samples analyzed for each ADAMTS enzyme is presented in Table 1.

### 3.3. Relationship Between VCAN Proteolysis and Desmoplasia

**Figure 6:**
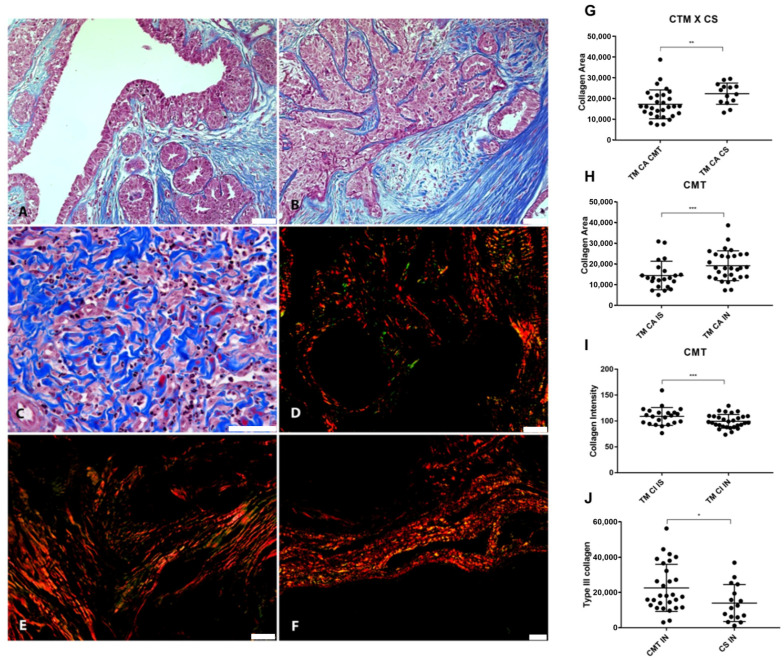
Relationship between VCAN proteolysis and desmoplasia. (**A**) Masson’s Trichrome staining revealing collagen deposition in the stroma adjacent to the in situ areas in CMT, 200×, bar 50 µm. (**B**) Masson’s Trichrome staining in the invasion areas in CMT, 200×, bar 50 µm. (**C**) Invasion areas in CSS stained with Masson’s Trichrome, 400×, bar 50 µm. (**D**) Picrosirius Red staining revealing the deposition of type I (in red) and type III (in green) collagen in the stroma adjacent to the in situ areas in the CMT, under polarized light, 200×, bar 50 µm. (**E**) Picrosirius Red staining in the areas of invasion in the CMT, under polarized light, 200×, bar 50 µm. (**F**) Picrosirius Red staining in the areas of invasion in the CSS, under polarized light, 100×, bar 50 µm. (**G**) Collagem area (CA) difference between CTM and CSS. (**H**) Collagen difference between in situ and invasion areas in CMT. (**I**) Collagen intensity (CI) on invasion and in situ area in CMT. (**J**) Type III collagen difference between CMT and CSS. Legend: *p*-value: * = <0.05; ** = <0.01; *** = <0.001.

The pattern of collagen fibers was analyzed using Masson’s Trichrome and Picrosirius Red in in situ and invasive areas to characterize the connective stroma adjacent to the areas of epithelial proliferation and to correlate it with the proteolysis of VCAN (Figure 6).

Our results showed that collagen areas were greater in CMT than in CSS (*p* = 0.0342) (Figure 6G). Areas of neoplastic invasion in the CMT tended to have a greater collagen area (*p* = 0.0003) (Figure 6H). However, the stroma adjacent to the in situ areas had more intensely stained collagen fibers in the CMT (*p* < 0.0001) (Figure 6I). Type III collagen was more abundant in CMT compared to CSS (*p* = 0.275) (Figure 6J). Inverse correlations were found between type III collagen and VCAN expression in carcinomatous cells from in situ areas (*p* = 0.0139, r = −0.4951).

## 4. Discussion

In female dogs, mammary tumors account for more than 50% of neoplasms, showing similarities to human breast cancer in terms of histopathology, therapeutic response, and molecular characteristics. These similarities make female dogs an ideal comparative model for studying breast cancer in women [25,26,27]. Deciphering the behavior of tumor cells allows for an understanding of the dynamics of the environment they inhabit. In this context, the extracellular matrix, which orchestrates cellular activity, functions as a network for connection and support. Under pathological conditions, ECM elements become regulated by tumor cells, leading to dysfunctional remodeling driven by proteolytic enzymes [1,4,5,22].

Although VCAN is considered one of the most studied ECM components in human breast cancer [3], little is known about the influence of its proteolysis on tumor progression. Some studies show that the bioactive fragments of VCAN (matrikines) generated by the action of the ADAMTS enzymes can contribute to regulating the inflammatory infiltrate and antitumor immune response in various neoplasms. VKINE, the best characterized of these proteolytes, is associated with the presence of intratumoral Batf3-DC cells and T-cell infiltration in several neoplasms, both solid and hematopoietic [41]. However, until now, the potential of VKINE has not yet been explored in the context of human breast cancer or in a mammary canine model.

In the present study, the immunohistochemical VCAN expression was observed in all evaluated samples of CMTs and CSS, with predominant expression in the peritumoral stroma. In CMTs, the prominent immunostaining of VCAN was observed in the stroma associated with invasion areas, corroborating the findings of DAMASCENO and collaborators [8], who also found an association between VCAN and invasiveness in CMT. Previously, RICCIARDELLI and collaborators [7], when analyzing VCAN in breast carcinomas of women, noticed that its expression was more evident and diffused around malignant areas, while the stroma surrounding areas considered benign showed negligible immunoreactivity for PG. In the CSS group, no difference was found in the VCAN expression between in situ and invasion areas, reinforcing the more aggressive nature of CSS, including expression in areas of neoplastic proliferation contained by the basement membrane, similar to observations in women [31,42].

Interestingly, in addition to the known stromal marking of its precursor VCAN, VKINE was strongly present in the cytoplasm of malignant epithelial cells. Specifically, 75.5% of the cases showed VKINE epithelial marking, while only 16.6% had epithelial VCAN expression. Moreover, no correlation was observed between the presence of VKINE and VCAN. Previous studies have also shown that VKINE may have a different location than VCAN. For example, McCulloch and collaborators [43] demonstrated that in embryonic interdigital tissue, VKINE was expressed in the cytoplasm of cells destined to undergo apoptosis for tissue regression. In contrast, the integral form of VCAN was minimally expressed in this region and limited to other areas of the limb. Another study detected a distinct and specific location of VKINE compared to VCAN, suggesting a process of relocating free VKINE into endothelial cells in a murine model of mammary cancer by mechanisms not yet understood [44]. These findings indicate that VKINE possibly assumes functions independent of the intact form of VCAN, raising hypotheses for its origin in the cytoplasm of neoplastic cells in our study. Neoplastic cells may be able to synthesize VKINE, or this matrikine could be relocated into the carcinomatous cells, as suggested by Asano and collaborators [44]. Considering that, to date, the only known way of generating VKINE is through VCAN proteolysis, the translocation of this stromal proteolyte into neoplastic cells is believed to be the most robust hypothesis.

Our results show that, like VCAN, VKINE was more expressed in epithelial cells from invasion areas in CMT. According to Timms and Maurice [45], the bioactivity of VCAN proteolytes is context dependent. In our analysis, VKINE was associated with invasion areas in a type of mammary tumor; however, further studies are needed to better understand its potential prognostic value and role in tumor progression. Dhakal and collaborators [46] evaluated VCAN proteolysis in the post-transplant survival of patients with myeloma and showed that the group with low VKINE expression had better overall survival and response rates. A recent study demonstrated that VCAN accumulation and low VKINE expression ameliorate acute colitis. Using a co-culture system, the authors showed that the overexpression of VKINE produced by macrophages inhibited the differentiation of fibroblasts into myofibroblasts, suggesting that VKINE acts as a direct regulator in the repair of inflamed tissue [47].

Little is known about the effects of VCAN cleavage, the functions of its fragments, or how this remodeling of the ECM affects the microenvironment and the phenotype of inflammatory or tumor cells present within it. Thus, in the present study, after evaluating VCAN and VKINE, we sought to characterize the expression of ADAMTS family enzymes and their relationship with PG proteolysis in canine tumors.

In the immunohistochemical evaluation of ADAMTS-1, cytoplasmic marking was observed in tumor cells and rarely in the stroma, corroborating the findings of Silva and collaborators [48], who also detected ADAMTS-1 expression in cancer cells, including in the nucleus. No nuclear staining was observed in the tumor cells of the analyzed samples. Additionally, most samples did not show expression of this enzyme, similar to the results of another research group, which showed that ADAMTS-1 is poorly expressed in human breast cancer (invasive ductal carcinoma) compared to normal breast samples [35]. In contrast, in the context of multiple myeloma, ADAMTS-1 was overexpressed and identified as one of the main versicanases [19].

To date, the literature suggests that ADAMTS-1 is the best-characterized tumor-promoting ADAMTS metalloprotease [49]. Its expression is associated with tumor development and increased risk of bone metastasis in women with breast cancer [50]. In this study, we were not able to observe a significant correlation between the presence of ADAMTS-1 and the VCAN expression or its proteolysis, indicating that this enzyme may not be primarily responsible for VCAN proteolysis, as shown by Santamaria and collaborators [51].

According to our findings, the ADAMTS-5 enzyme showed no significant correlation with VCAN and VKINE expression in the canine mammary tumor subtypes studied. Although Santamaria and collaborators [51] concluded in their studies with cell lines (HEK293 EBNA expressing ADAMTS-4 and -5) that ADAMTS-5 is a versicanase 18 times more potent than ADAMTS-4 (while the activity of the versicanase ADAMTS-1 is comparatively low), this enzyme may exhibit different behaviors in mammary tumors. Further studies employing new strategies for qualitative and quantitative analysis of ADAMTS-15 expression are needed, particularly with a larger sample size and a broader range of tumor subtypes beyond mixed tumors.

Despite ADAMTS-5 showing no correlation with VKINE, this enzyme exhibited a strong expression in both CMT and CSS. In CSS, epithelial expression was particularly evident, both in in situ areas and in regions of neoplastic invasion, suggesting a possible association with the greater aggressiveness of this tumor. This finding aligns with observations made by Haraguchi and collaborators [52] in colorectal cancer.

Recently, Barallobre-Barreiro and collaborators [10] showed in animal models and patients that ADAMTS proteases, especially ADAMTS-5, are critical for VCAN degradation in the heart and that VCAN accumulation is associated with impaired cardiac function. However, this enzyme had no significant correlation with VCAN expression or VKINE. Little is known about the role of ADAMTS-5 in breast cancer; however, recently, a study suggested that its low expression in this tumor type in women was associated with late-onset tumors (≥55 years) [53].

ADAMTS-8 is not commonly related to the presence of VKINE; we noted that the expression of ADAMTS-8 in invasion areas is inversely proportional to the expression of VKINE in those same areas. The epithelial expression of ADAMTS-8 was higher in CMT compared to CSS; that is, ADAMTS-8 stood out in the tumor with its better prognosis, in addition to being strongly expressed in most of the studied samples, and was described in the literature in the study of other breast, brain, gastric, and pancreatic tumors in humans [54,55]. ADAMTS-8 and ADAMTS-9 were also expressed in the cases studied, but there was no correlation between the immunostaining of this ADAMTS and VCAN degradation.

Many studies associate ADAMTS-9 with VCAN proteolysis in different scenarios, such as embryogenesis [12] and cardiac and aortic anomalies [56]. However, there is little information about the activity of ADAMTS-9 versicanase in the tumor microenvironment. ADAMTS-9 was also expressed in the present study, but there was no correlation between the immunostaining of this ADAMTS and VCAN degradation. Although ADAMTS-9 has been linked to VCAN proteolysis in various contexts, including embryogenesis [12] and cardiac and aortic anomalies [56], its role in the tumor microenvironment remains underexplored.

Interestingly, invasion areas demonstrated a notably low expression or absence of ADAMTS-9 marking in the stroma. In addition, when comparing CMT and CSS, the relationship between ADAMTS-9 and less aggressive tumor behavior becomes more evident. CTM exhibited higher amounts of ADAMTS-9 compared to CSS. This result aligns with findings in human breast carcinoma cell lines, where ADAMTS-9 has been downregulated in malignant and invasive breast carcinomas compared to non-neoplastic ones [57].

In the cases studied, ADAMTS-15 was the only enzyme that showed a correlation with VCAN proteolysis. This finding aligns with Dancevic and collaborators [58], who characterized ADAMTS-15 as a new VCAN proteoglycanase that likely acts in synergy with other members of the ADAMTS family during initial cardiac development and musculoskeletal development in mice. In the present study, the epithelial labeling of ADAMTS-15 was higher in CMT, a tumor with a better prognosis, compared to CSS, which has a worse prognosis. In another study, ADAMTS-15 expression was correlated with improved prognosis in women with breast cancer, reduced motility of breast cancer cell lines, and decreased tubule formation in endothelial cell lines [57]. In human prostate cancer, it has been observed that ADAMTS-15 was expressed in biopsies, with evidence of co-localization with VCAN and its bioactive cleavage fragment, versikine, suggesting a tumor suppressor role for ADAMTS-15 in prostate cancer [11]. In this study, ADAMTS-15 was more evident in stromal areas of CSS than in stromal areas of CMT, indicating that further studies are needed to understand the role of ADAMTS-15 and its localization in cancer.

As the ECM is a multitasking agent in cancer dynamics, collagen fibers from areas in situ and invasion were also characterized to better investigate the remodeling of the ECM against VCAN proteolysis in the tumor microenvironment, correlating them to the presence of VKINE. It is known that small PGs rich in leucine (SLRPs), including decorin and lumican, are mediators of fibrillogenesis and collagen organization. However, the role of large PGs like VCAN in remodeling collagen fibers is still poorly understood.

A recent study has shown that staining collagen fibers with Picrosirius Red under polarized light can be an excellent routine method for assessing collagen signatures in human breast cancer and even replace more robust methods such as second-harmonic generation microscopy [40]. In our study, type III collagen (green fibers under polarized light) was more abundant in CMT compared to CSS. Type III collagen may be associated with better prognosis tumors because these fibers drive stromal organization and limit metastasis, as demonstrated in a murine model of mammary cancer [59].

Inverse correlations were found between type III collagen and VCAN expression in carcinomatous cells from in situ areas. The negative correlation between VCAN and type III collagen may be associated with the potential of VCAN to induce fiber compaction. Thus, VCAN would be more related to more compact fibers, such as type I collagen, rather than looser fibers like collagen III [60]. We showed that the group with high VKINE expression had smaller areas of type III collagen fibers compared to the group with low expression. However, so far, no studies have evaluated the relationship between VCAN proteolysis and the deposition and classification of collagen fibers. It is known that VCAN is an important agent in collagen fibrillogenesis, and its degradation can influence the reorganization of collagen fibers in the tumor microenvironment. According to Brissom and collaborators [59], type III collagen mechanically suppresses the pro-carcinogenic microenvironment by regulating stroma organization, including the density and alignment of fibrillar collagen and myofibroblasts. Thus, VKINE may be involved in reduced deposition of type III collagen, contributing to a microenvironment favorable to invasion.

## 5. Conclusions

Our study suggests, for the first time, in the context of mammary cancer in a canine model, that VCAN proteolysis is an important player in the tumor microenvironment, associated with invasiveness and reduced deposition of type III collagen. These findings support further investigation of VKINE to better understand the dynamics occurring in the ECM during cancer progression. This research could lead to the development of new diagnostic and therapeutic tools for both human and canine mammary tumors.

## Figures and Tables

**Table 1:** Median ADAMTS expression in in situ and invasive carcinomatous cells, as well as in stromal areas adjacent to CMT and CSS.

	Epithelium			Stroma		
	IS	IN		IS	IN	
	Median (n/Total)	Median (n/Total)	*p* Value	Median (n/Total)	Median (n/Total)	*p* Value
ADAMTS-1	0 (47/47)	0 (39/47)	0.8532	0 (37/47)	0 (39/47)	0.5000
ADAMTS-5	145 (26/47)	120 (32/47)	0.0879	0 (21/47)	0 (23/49)	>0.9999
ADAMTS-8	60 (21/47)	70 (31/47)	0.2345	0 (19/47)	0 (30/47)	>0.9999
ADAMTS-9	35 (24/47)	50 (33/47)	>0.9999	0 (19/47)	0 (28/47)	-
ADAMTS-15	120 (13/47)	120 (42/47)	0.4609	10 (15/47)	20 (43/47)	0.7500

Legend: IS = In situ; IN = invasive. Wilcoxon’s test, 95% confidence interval. *p*-values were considered significant when less than 0.05.

## Data Availability

The data presented in this study are available in this article and Supplementary Materials.

## Supplementary Materials

The following supporting information can be downloaded at: https://www.mdpi.com/article/10.3390/cancers16234057/s1, Table S1: Median of ADAMTS immunohistochemical expression in invasive carcinomatous and in situ areas in both epithelium and stroma of CSS; Table S2: Median of ADAMTS immunohistochemical expression in invasive carci-nomatous and in situ areas in both epithelium and stroma of CMT.
