# Supplementary material for: Versican Proteolysis by ADAMTS: Understanding Versikine Expression in Canine Spontaneous Mammary Carcinomas
Source: Cancers (Basel). 2024 Dec 4;16(23):4057. doi: 10.3390/cancers16234057 (PMC11640010; doi:10.3390/cancers16234057)
Supplement: Supplementary file 1 [file cancers-16-04057-s001.zip › cancers-3173973-supplementary.pdf]

Table S1: Median of ADAMTS immunohistochemical expression in invasive carcinomatous and in situ areas in both epithelium and stroma of CSS

	Epithelium			Stroma		
	**IS**	**IN**		**IS**	**IN**	
	**Median (n/total)**	**Median (n/total)**	***P value***	**Median (n/total)**	**Median (n/total)**	***P value***
ADAMTS-1	0 (17/17)	0 (11/17)	*0,8750*	0 (11/17)	0 (11/17)	-
ADAMTS-5	60 (07/17)	90 (08/17)	*>0,999*	0 (06/17)	0 (05/17)	-
ADAMTS-8	20 (05/17)	50 (09/17)	*0,500*	0 (05/17)	0 (09/17)	*>0,999*
ADAMTS-9	10 (06/17)	30 (11/17)	*0,500*	0 (05/17)	0 (08/17)	-
ADAMTS-15	20 (01/17)	105 (16/17)	-	5 (01/17)	55 (16/17)	-

Wilcoxon's test, 95% confidence interval. P-values were considered significant when less than 0,05.

Table S2: Median of ADAMTS immunohistochemical expression in invasive carcinomatous and in situ areas in both epithelium and stroma of CMT

	Epithelium			Stroma		
	**IS**	**IN**		**IS**	**IN**	
	**Median (n/total)**	**Median (n/total)**	***P value***	**Median (n/total)**	**Median (n/total)**	***P value***
ADAMTS-1	0 (30/30)	0 (28/30)	*0,8281*	0 (26/30)	0 (28/30)	*0,500*
ADAMTS-5	200 (19/30)	150 (24/30)	*0,07*	0 (15/30)	0 (18/30)	-
ADAMTS-8	80 (16/30)	80 (22/30)	*0,6646*	0 (14/30)	0 (21/30)	*>0,999*
ADAMTS-9	55 (18/30)	55 (22/30)	*0,5801*	0 (14/30)	0 (20/30)	-
ADAMTS-15	125 (12/30)	147,5 (26/30)	*0,4609*	12,5 (14/30)	15 (27/30)	*0,7500*

Wilcoxon's test, 95% confidence interval. P-values were considered significant when less than 0,05.
